# Author Correction: Slab tearing and its surface signals controlled by passive margin strength

**DOI:** 10.1038/s41467-026-74788-1

**Published:** 2026-07-07

**Authors:** Giridas Maiti, Nevena Andrić-Tomašević, Attila Balázs, Lucas H. J. Eskens, Taras Gerya

**Affiliations:** 1 Institute of Applied Geosciences, Karlsruhe Institute of Technology, Karlsruhe, Germany; 2 Institute of Geophysics, ETH, Zürich, Switzerland

**Keywords:** Geodynamics, Tectonics, Sedimentology

Correction to: *Nature Communications* 10.1038/s41467-026-73963-8, published online 04 June 2026

In the version of this article initially published, due to a file conversion error, in Fig. 1a, black trace lines for collision boundaries in the Carpathians and Apennines were disconnected from their respective arrow points. The figure is now amended in the HTML and PDF versions of the figure.

