# Supplementary material for: Slab tearing and its surface signals controlled by passive margin strength
Source: Nat Commun. 2026 Jun 4;17:4964. doi: 10.1038/s41467-026-73963-8 (PMC13236983; doi:10.1038/s41467-026-73963-8)
Supplement: Supplementary file 1 — Supplementary Information [file 41467_2026_73963_MOESM1_ESM.pdf]

Supplementary Information for

Slab tearing and its surface signals controlled by passive margin strength

Giridas Maiti*[1], Nevena Andrić-Tomašević[1], Attila Balázs[2], Lucas H. J. Eskens[1], Taras Gerya[2],
[1]Institute of Applied Geosciences, Karlsruhe Institute of Technology, Germany
[2]Institute of Geophysics, ETH Zürich, Switzerland
***Corresponding author: Giridas Maiti (giridas.maiti@kit.edu)

Content:

Table S1-S3

Figures S1- S25

Table S1: Model parameters

Parameters	***Values***
Horizontal model dimensions	1800 × 800 km^2
Vertical model dimension	690 km
Temperature at the base of the lithosphere	1330 °C
Heat capacity, C_p (J/kg K)	1000
Thermal expansion, α' (1/K)	3 × 10^{-5}
Push velocity at the continent on the right side	3 – 0.75 cm yr^{-1}
Diffusion coefficient for erosion and sedimentation	0.5, 1, 2 km^2/ ka

Parameters	***Upper cont. crust***	***Lower cont. crust***	***Lithospheric mantle***	***Basalt***	***Gabbro***	***Weak zone***	***Sediments***
Thickness (km)	20	18	76	2	5	-	varies
Rheology	wet quartzite	plagioclase	dry olivine	plagioclase	plagioclase	wet olivine	wet quartzite
Density, ρ_0 (kg m^{-3})	2750	3000	3300	3000	3000	3200	2600
Pre-exponential factor, $1/A_D$ (Pa^n s)	1.97 × 10^{17}	4.8 × 10^{22}	3.98 × 10^{16}	4.8 × 10^{22}	4.8 × 10^{22}	5.0 × 10^{20}	1.97 × 10^{17}
Activation energy, E (kJ mol^{-1})	154	238	532	238	238	470	154
Activation volume, (J mol^{-1} MPa^{-1})	0	0	1.20	0	0	1.20	0
Power law exponent, n	2.3	3.2	3.5	3.2	3.2	4.0	2.3
Cohesion (Pa)	3 ×10^6	3 ×10^6	3 ×10^6	3 ×10^6	3 ×10^6	3 ×10^6	3 ×10^6
Coefficient of friction, sin(φ)	0.3-0.1	0.3-0.1	0.6-0.0	0.1	0.3-0.1	0.1	0.1
Radioactive heat production, H_r (μW/m^3)	2	0.2	0.022	0.022	0.024	0.022	2

Table S2: Influence of along-strike passive-margin strength heterogeneities

Numerical models	***Initial thermal age of oceanic plate adjacent to the passive margin (Myr)***		***Thermal age difference of oceanic plate (Myr)***	***Presence of micro-continent***	***Tear start and end time (Myr)***	***Tear velocity (cm/yr)***
	Front side	***Rear side***				
Reference model (Model_1)	10	***50***	40	Yes	~20 to 27.6	10.5
Model_2	10	***40***	30	Yes	~20 to 25	16
Model_3	***20***	***20***	0	Yes	~20 to 23	26.6
Model_4	***40***	***40***	0	Yes	~22.8 to 36.2	5.97
Model_5	10	40	30	***No***	~27.6 to 32.2	17.39
Model_6	10	***10***	0	***No***	~20 to 20	instantaneous

Table S3: Rheological influence of mantle on tearing

Numerical models	***Ductile viscosity of lithospheric mantle***	***Plastic viscosity (*****frictional coefficient*****) of lithospheric mantle***		***Tear start and end time (Myr)***	***Tear velocity (cm/yr)***
	Activation volume	**Initial (*b0*)**	**Final (*b1*)**		
Model_1	1.20	0.6	0.0	~20 to 27.5	10.5
Model_7	1.20	0.6	***0.2***	~20 to 28.9	8.98
Model_8	1.20	***0.3***	0.0	~17 to 20	26
Model_9	***1.50***	0.6	0.0	~25 to 42	4.7
Model_10	***1.75***	0.6	0.0	~30 to 40 and then stop	4

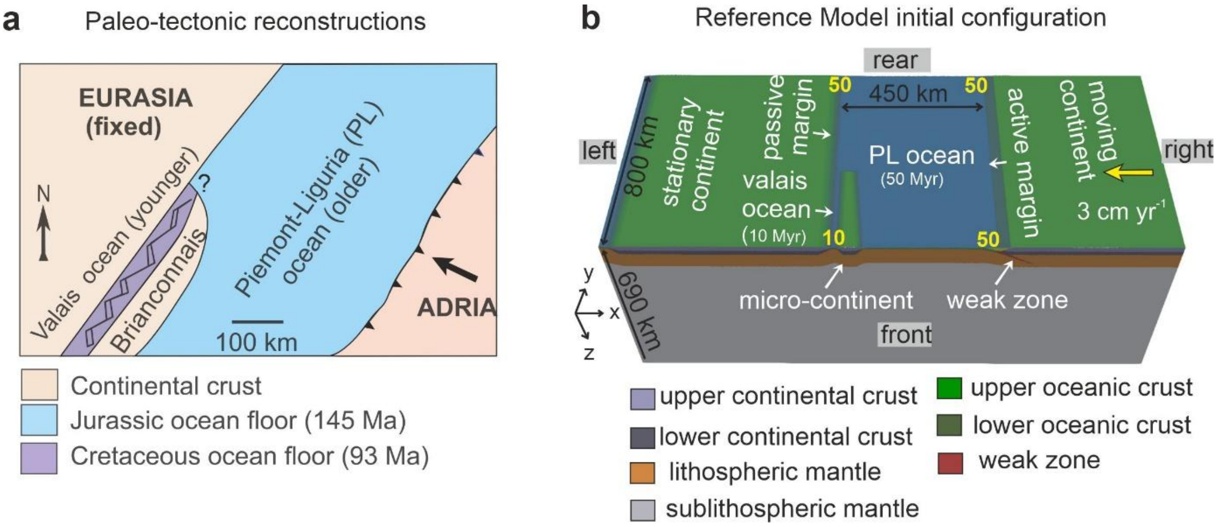

Fig. S1. Paleo-tectonic reconstructions map and initial configuration of the reference model. a, Palaeotectonic reconstruction at ~83 Ma, prior to the Adria–Eurasia collision. The reconstruction is compiled based on refs.[39,67]. The question mark indicates that the exact paleo-location of the extent of Briançonnais is poorly constrained. **b**, Reference model initial setup includes a microcontinent and along-strike age difference of ocean-floor adjacent to the continental passive margin. The ages of the oceanic domains increase linearly from the frontal to the rear end of the passive margin. We implemented this by defining the oceanic ages at the four corners of the oceanic domain (shown in bold yellow colour).

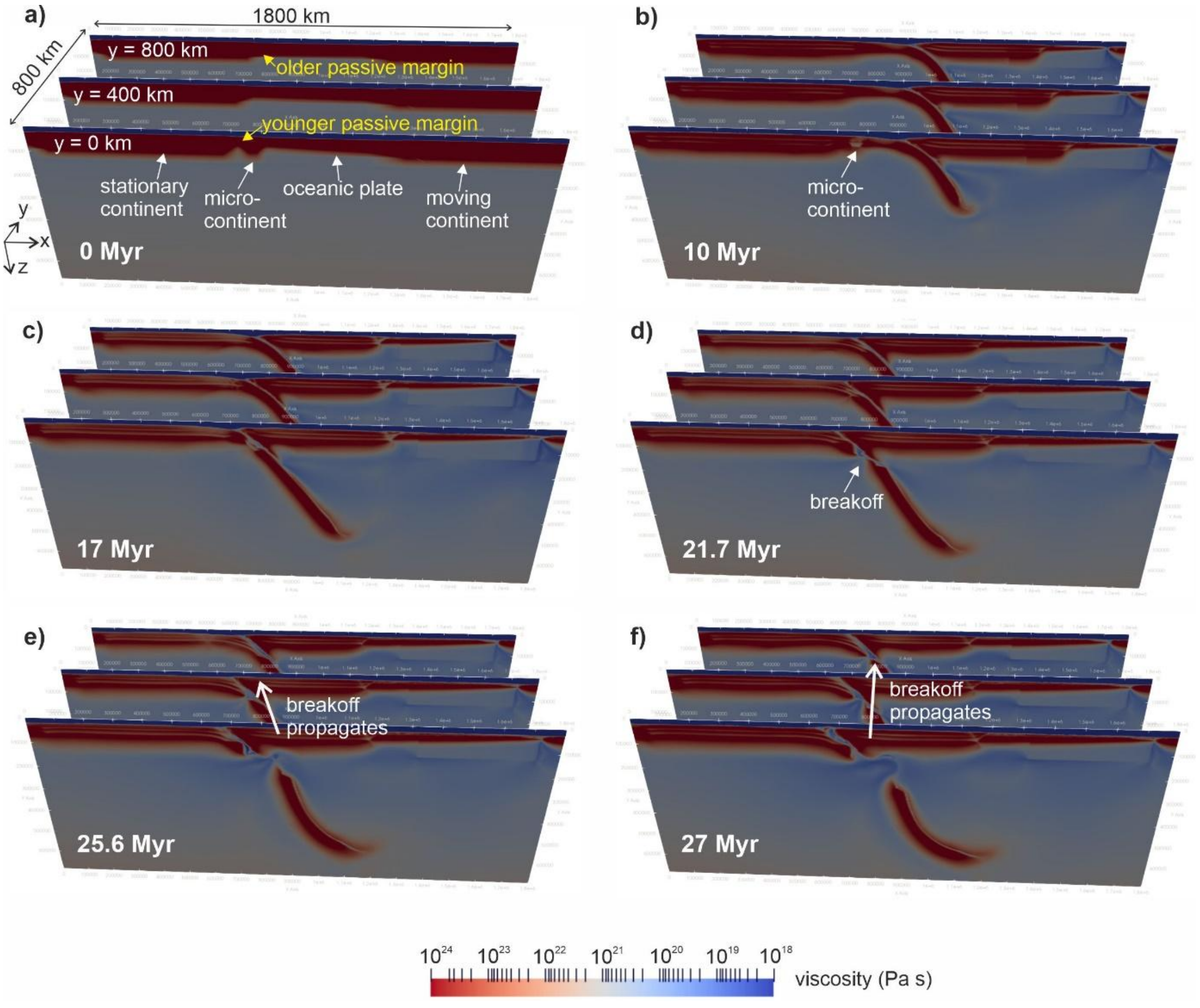

Fig. S2 Evolution of effective viscosity of subducting lithosphere in the reference model. In the initial model we have an along-strike difference oceanic plate adjacent to the passive margin. The passive margin age at the frontal section (y = 0 km) is 10 Myr, whereas at the rear side it is 50 Myr. This creates an along-strike difference in effective viscosity of oceanic lithosphere.

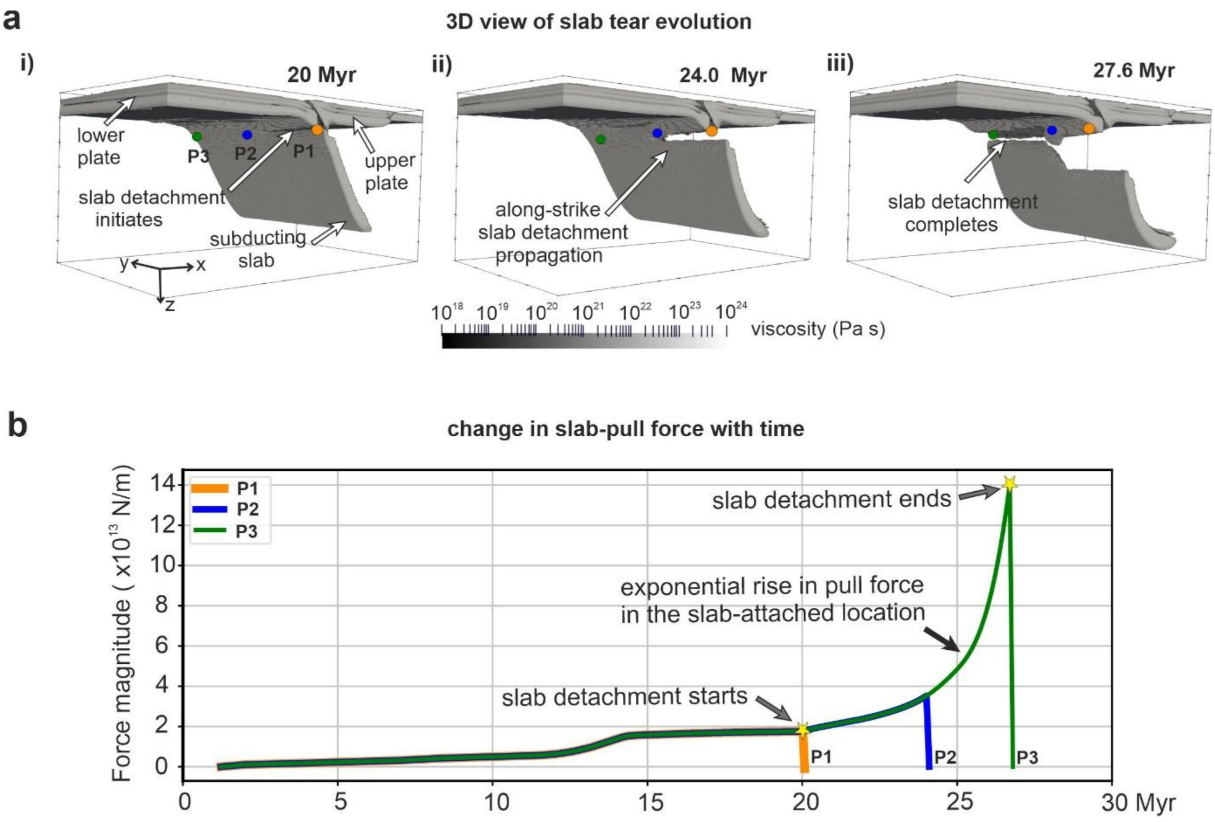

Fig. S3. **Change in slab pull forces during slab tear propagation measured at three different points**. **a**, Shows slab tear propagation in the lithosphere. **b**, shows how slab pull forces increases from points P1, to P2, and to P3 (marked in panel-a) as the slab tear propagates along-strike.

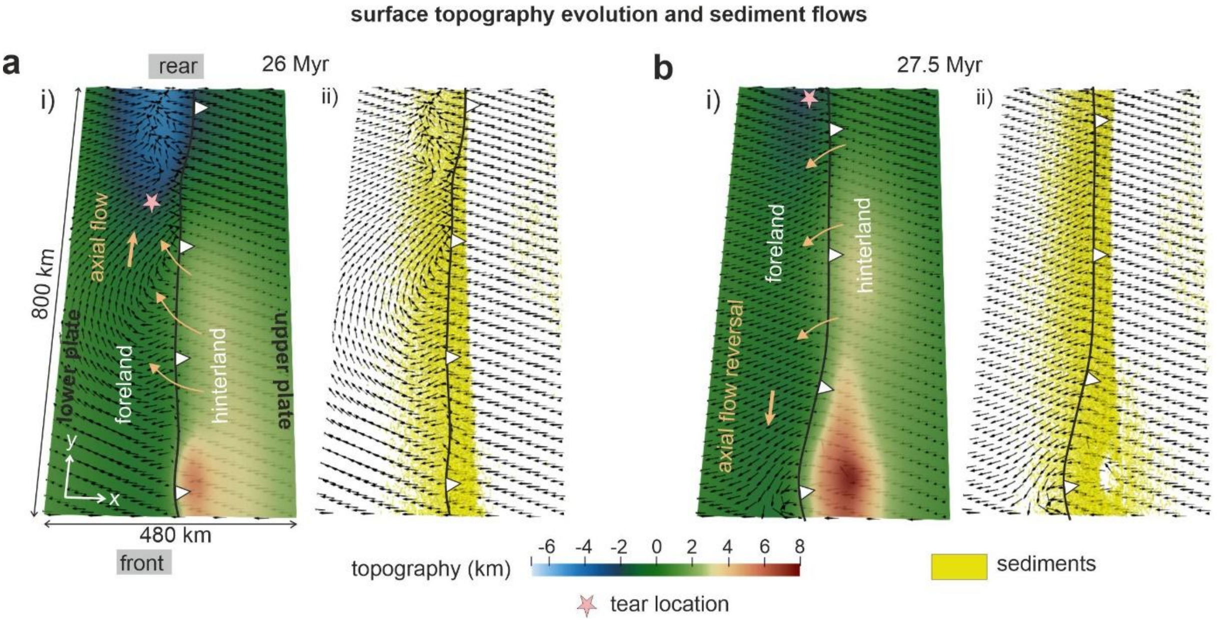

Fig. S4 Surface topography and flow of sediments in the foreland basins. **a**, Shows sediment flow from frontal end towards rear end. **b**, Shows sediment flow from rear end to frontal end.

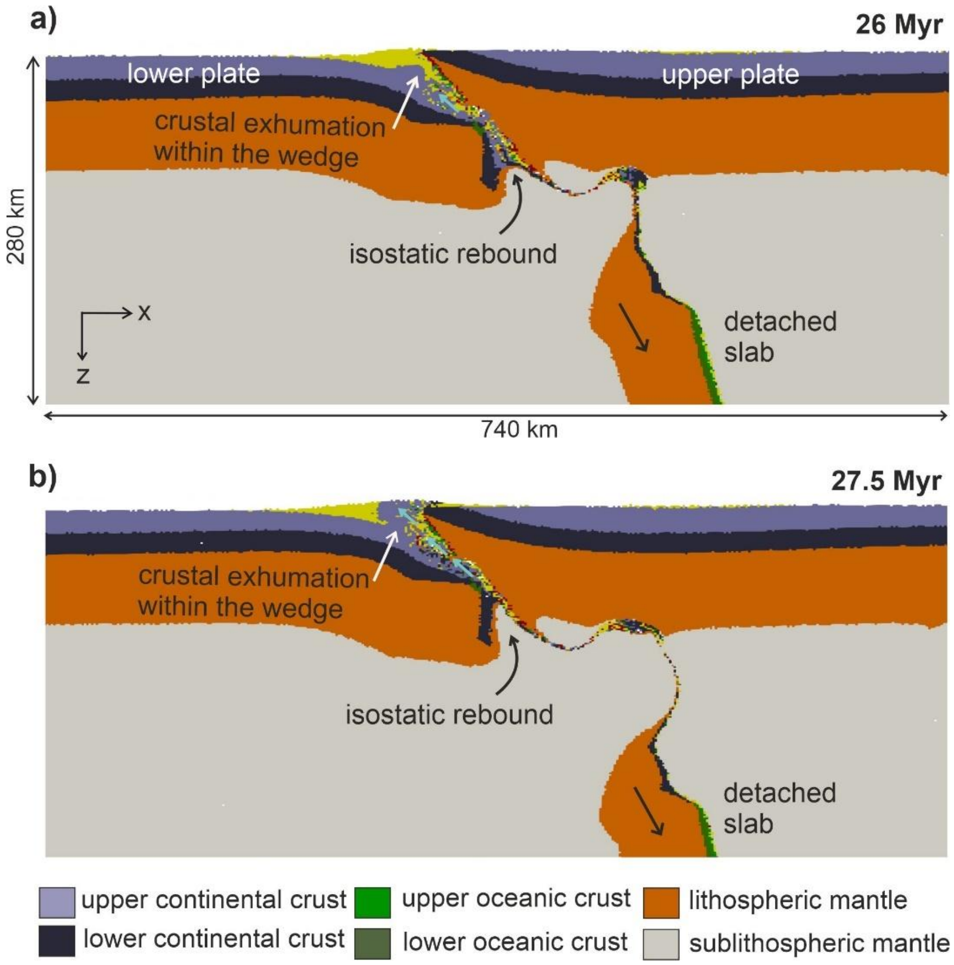

Fig. S5 Exhumation of deep-crustal rocks within the wedge. **a**, Upward exhumation of subducted upper continental crust and fragments of oceanic upper and lower crustal rocks within the wedge, primarily driven by the buoyancy of the subducted crust and isostatic rebound of the lower plate. **b**, Exhumation leads to the incorporation of deeply buried crustal rocks into the upper part of the orogenic wedge, with some reaching to the surface through thrusting and erosion, contributing to the high hinterland topography (Fig. 2e).

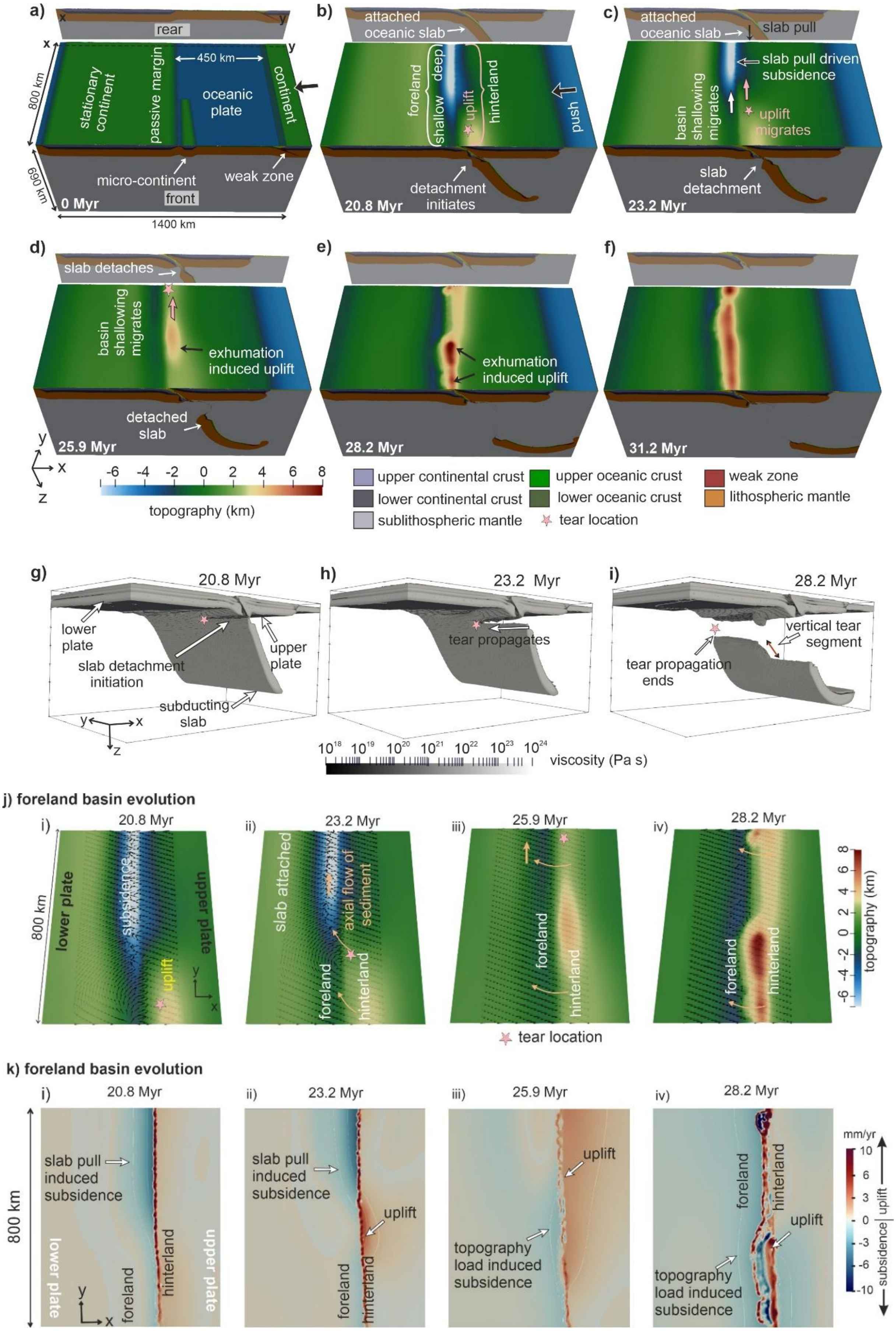

Fig. S6. **Evolution of Model_2**. Reducing the along-strike oceanic age difference from 40 Myr in the reference model to 30 Myr increases the tear velocity to 16 cm yr^{-1}.

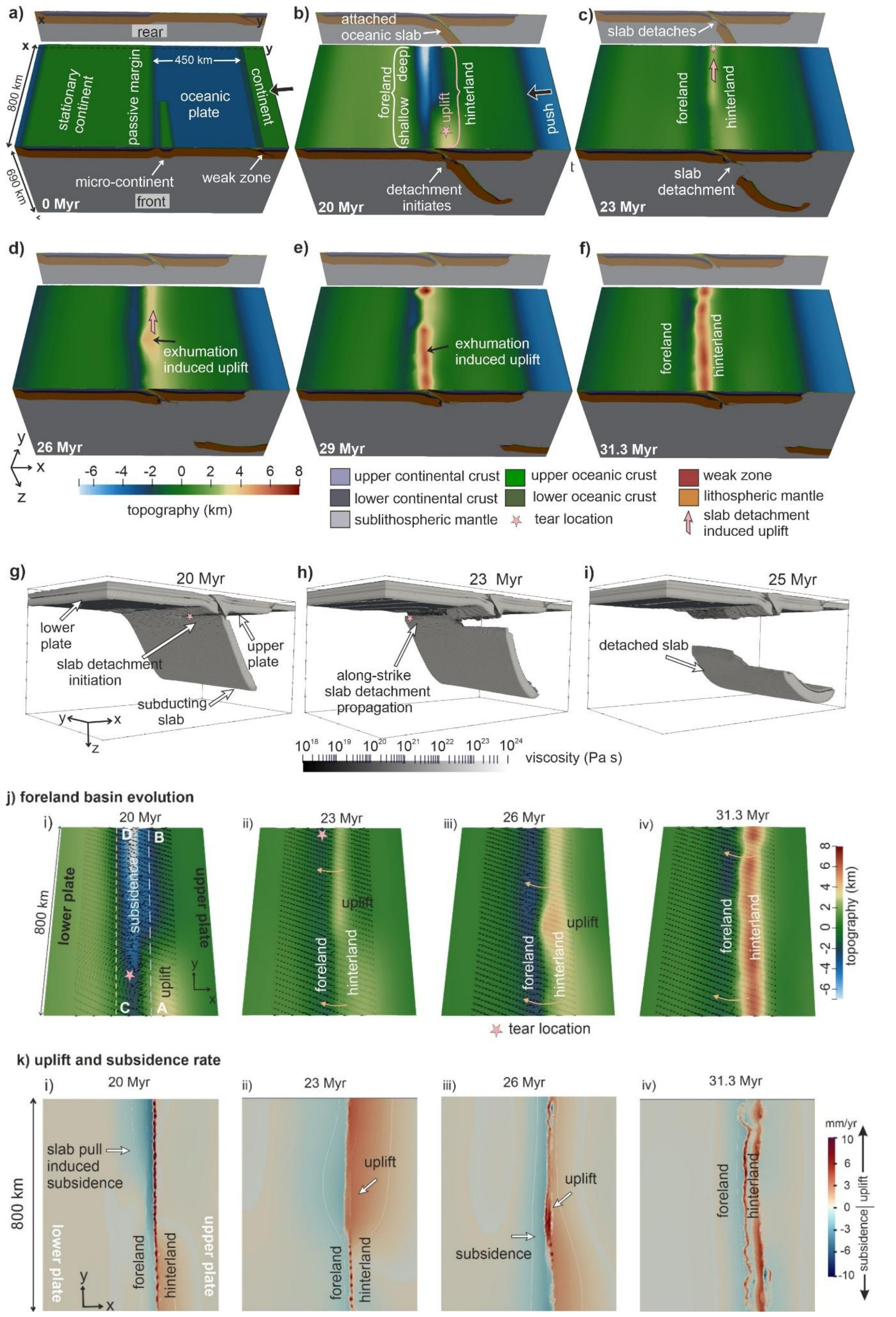

Fig. S7. Evolution of Model_3. This shows that a younger oceanic slab (20 Myr) with no along-strike age difference leads to rapid tear propagation at 26.6 cm yr^{-1}. Faster tearing shortens duration of differential uplift–subsidence and limits slab tearing surface expression in the foreland basin.

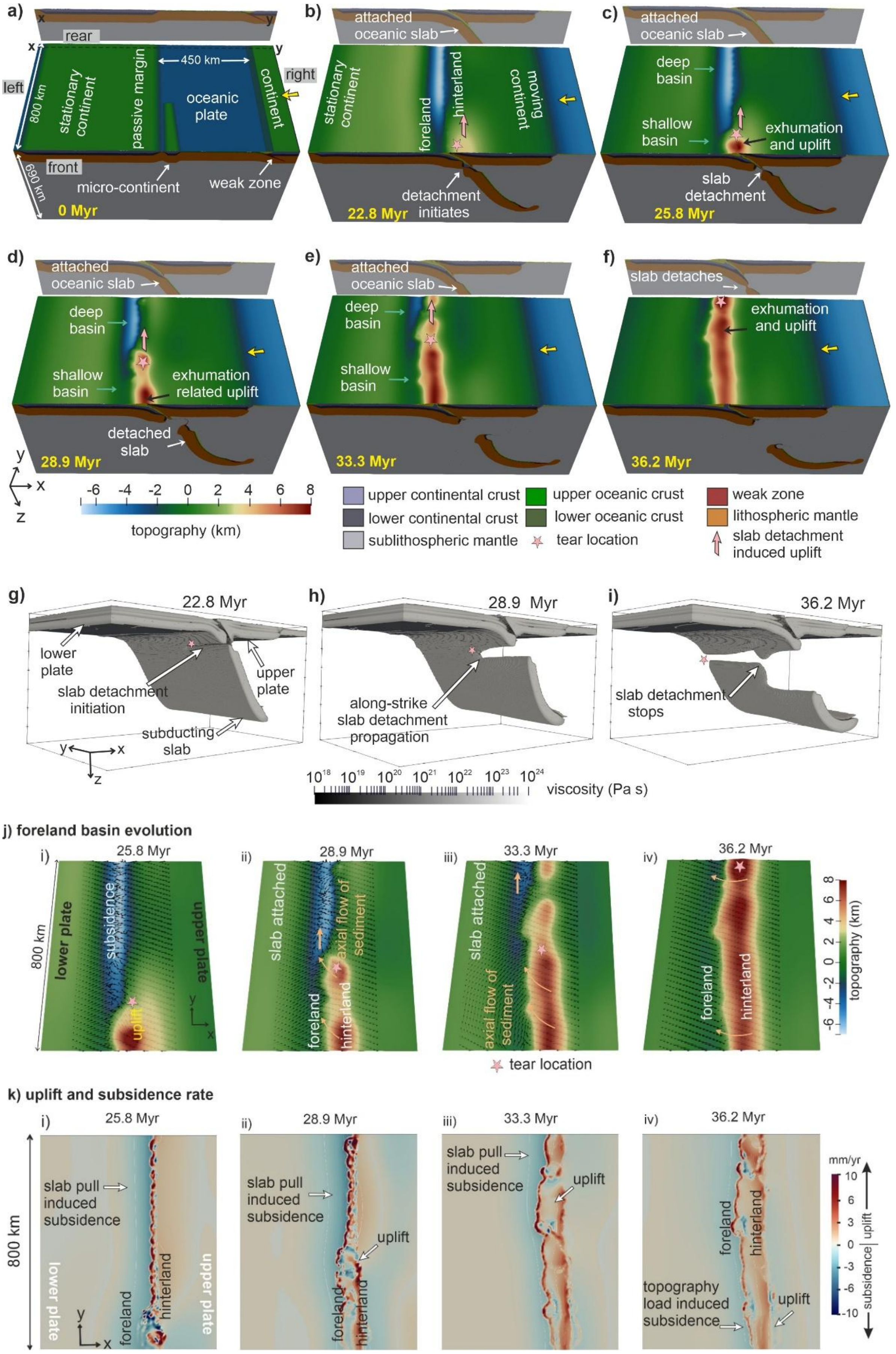

Fig S8. **Evolution of Model_4.** This shows that in an oceanic slab with no along-strike age difference, tearing can initiate due to the presence of a microcontinent, but after initiation it propagates slowly (~6 cm yr^{-1}) because of the stronger oceanic slab strength (40 Myr).

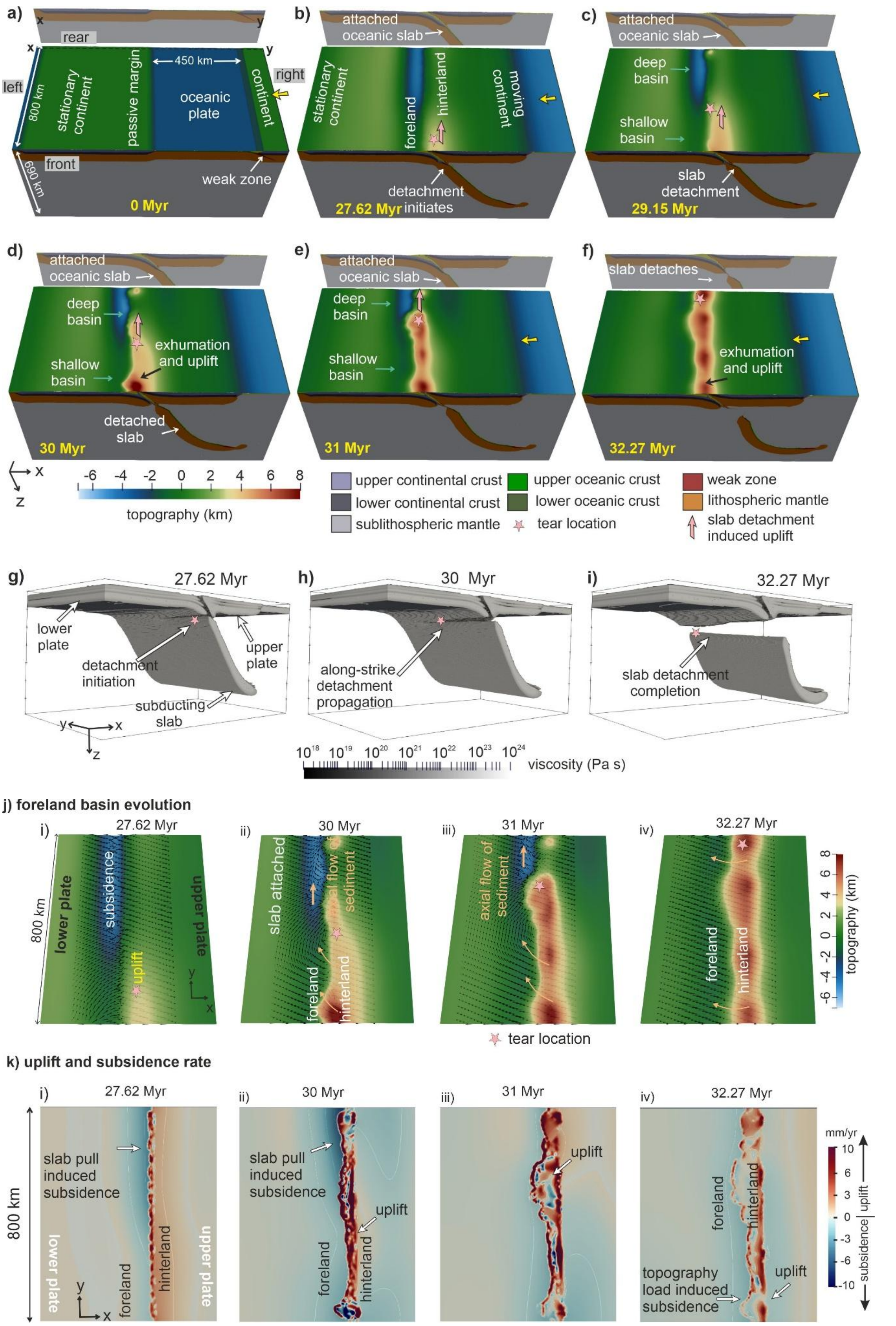

Fig S9. **Evolution of Model_5**. Removing the microcontinent but retaining a 30 Myr along-strike age contrast yields a comparable tear propagation velocity (17.4 cm yr⁻¹), indicating that the microcontinent in Model_2 slightly slows (16 cm yr⁻¹) tearing by inducing a minor vertical component.

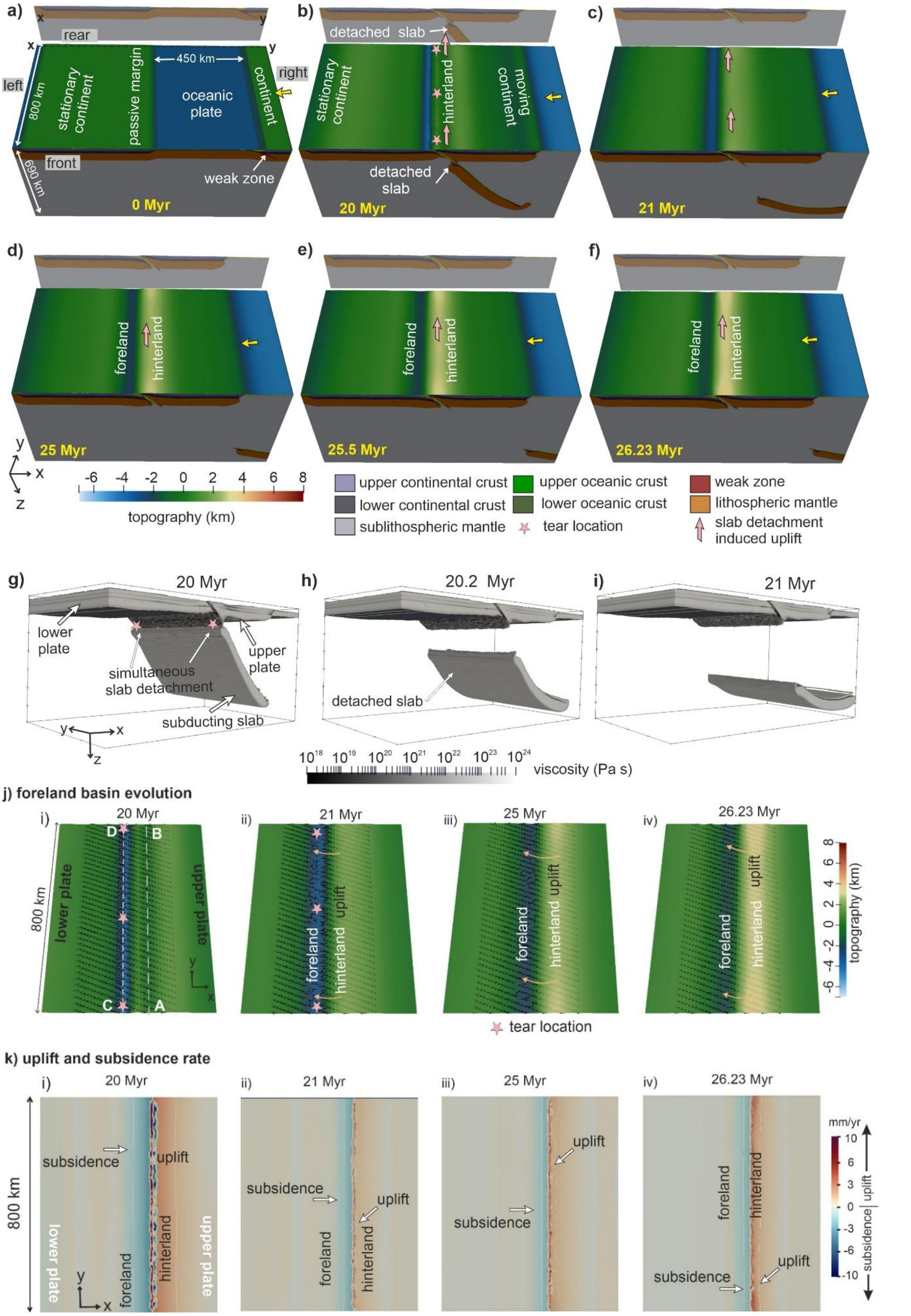

Fig S10. **Evolution of Model_6**. This shows in a homogeneous young oceanic slab (10 Myr slab age) with no microcontinent near passive margins, tearing occurs almost instantaneously. Such rapid tearing greatly shortens the duration of differential uplift–subsidence and produces a symmetric foreland basin architecture during tear propagation.

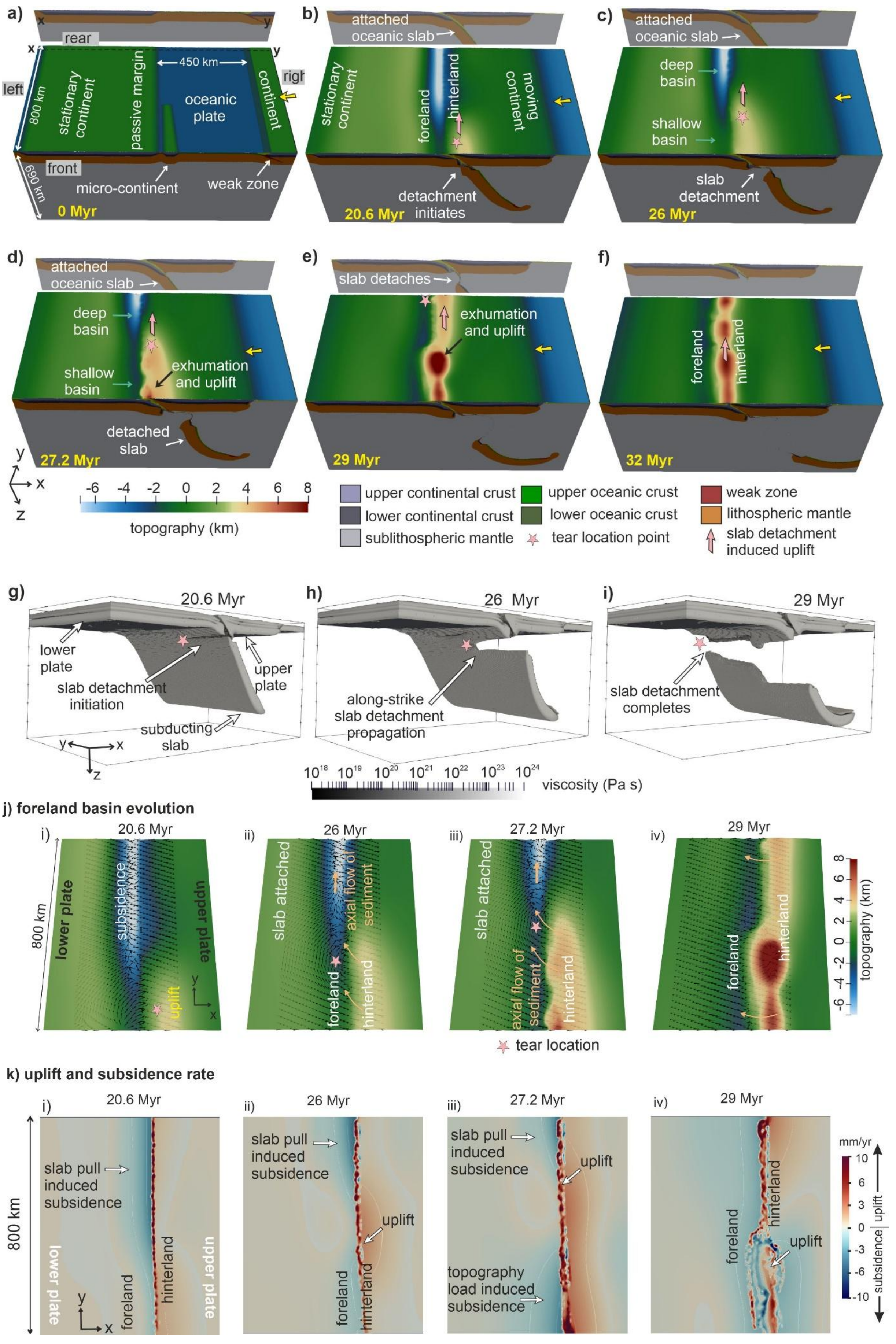

Fig S11. **Evolution of Model_7**. Increasing brittle frictional resistance (see Table S2) in the reference model (Model_1) slows tear propagation from 10.5 to 8.98 cm yr⁻¹.

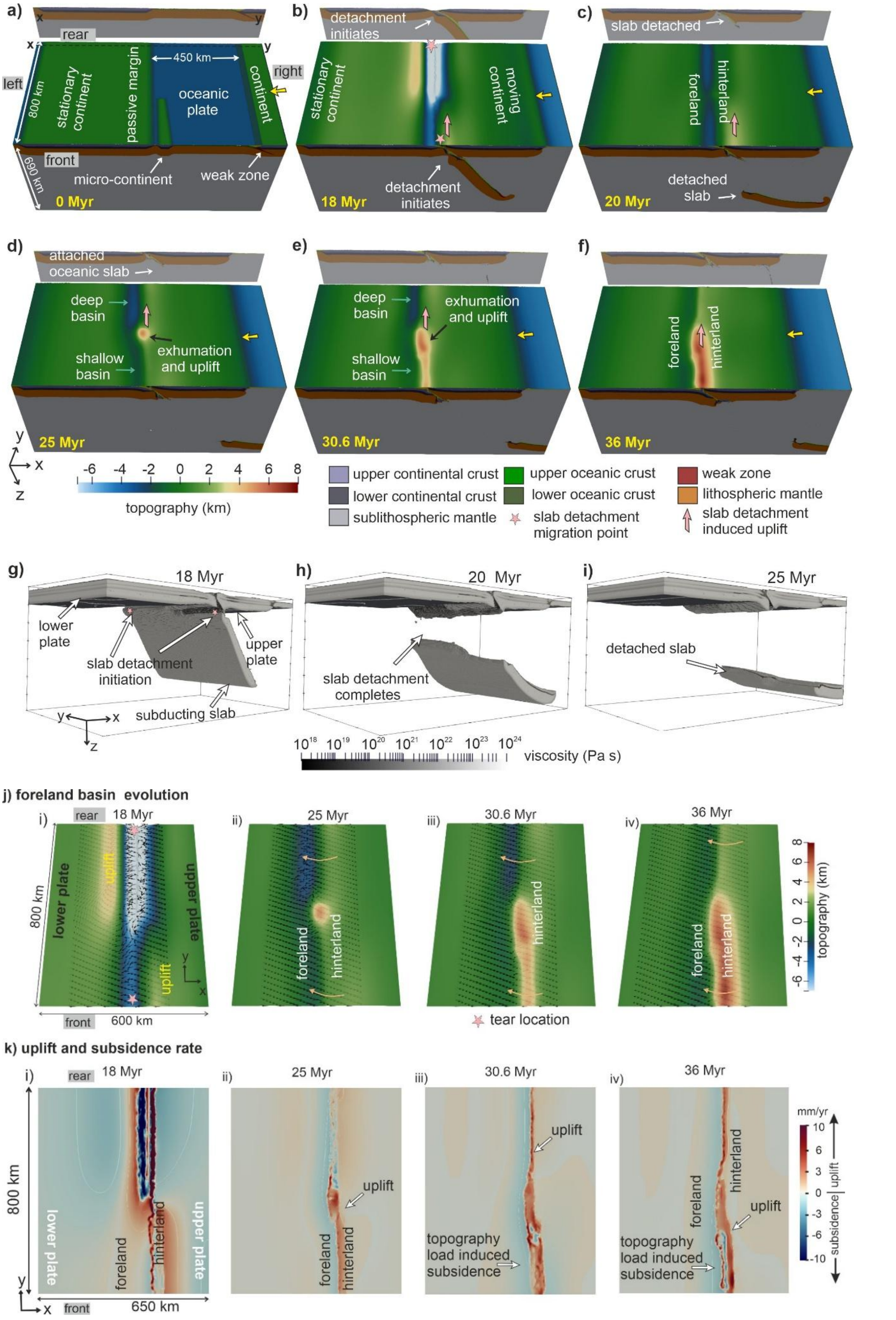

Fig S12. **Figure S8. Evolution of Model_8**. Lowering brittle frictional resistance (see Table S2) in the reference model (Model_1) increases tear propagation from 10.5 to 26 cm yr^{-1}. Faster tearing shortens duration of differential uplift–subsidence and limits slab tearing surface expression in the foreland basin.

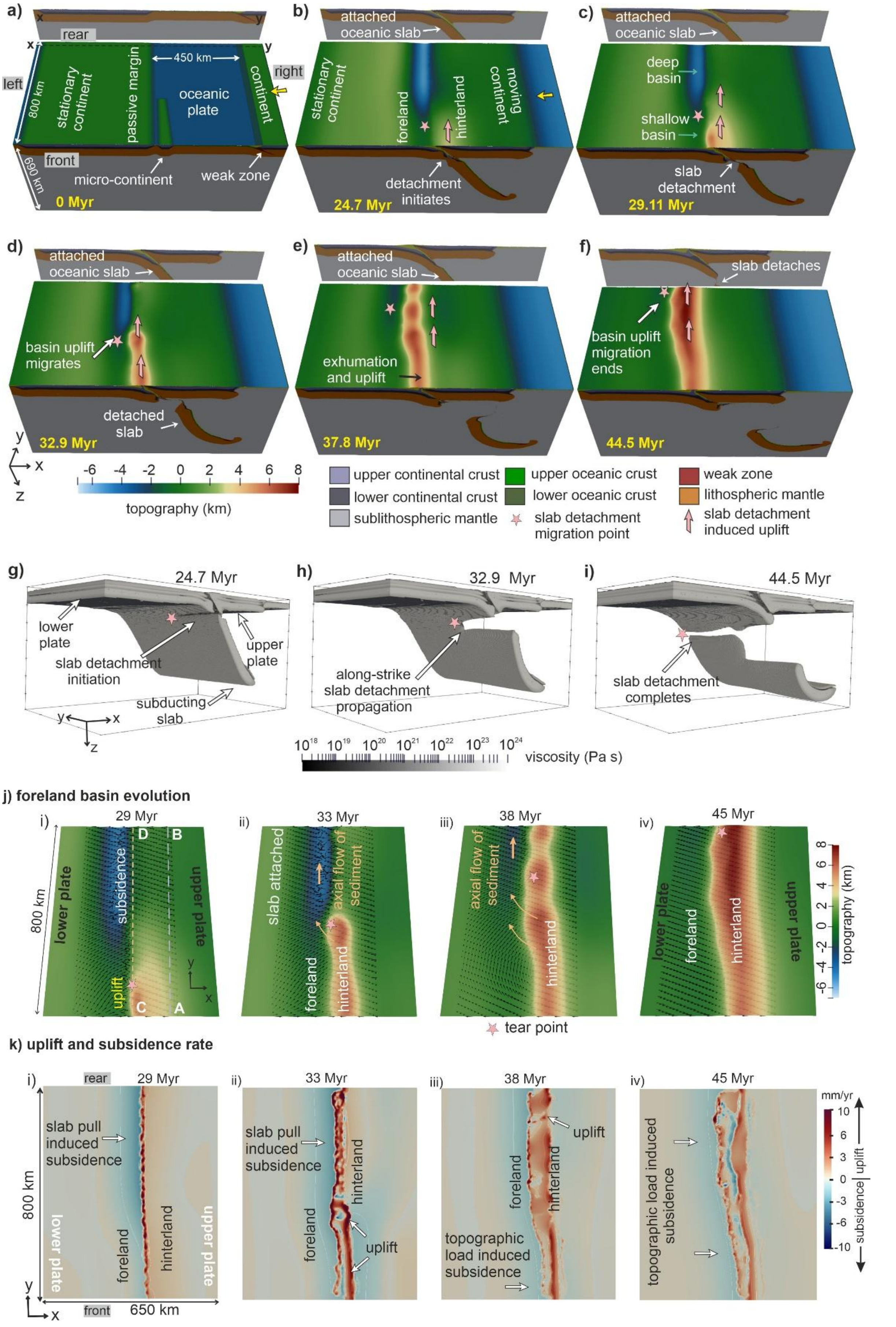

Fig S13. **Evolution of Model_9**. In the reference model (Model_1), increasing the ductile viscosity (see Table S2) of the subducting lithosphere by raising the activation volume slows tear propagation from 10.5 to 4.7 cm yr^{-1}.

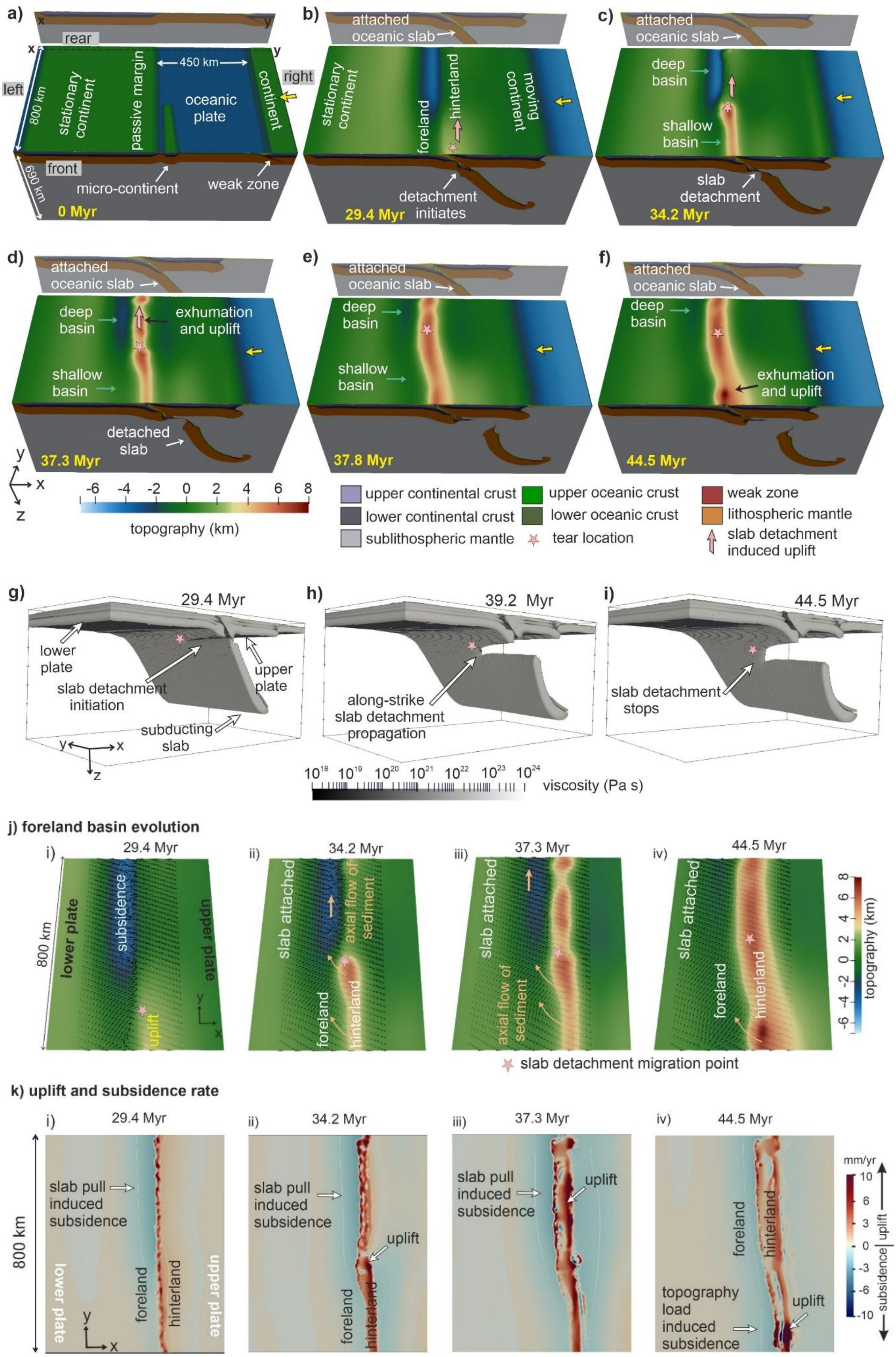

Fig S14. **Evolution of Model_10**. In the reference model (Model_1), increasing ductile strength (see Table S2) significantly by raising the activation volume causes lateral tearing to propagate very slowly. The tear initiates in the microcontinent region, but the stronger ductile lithosphere resists along-strike propagation.

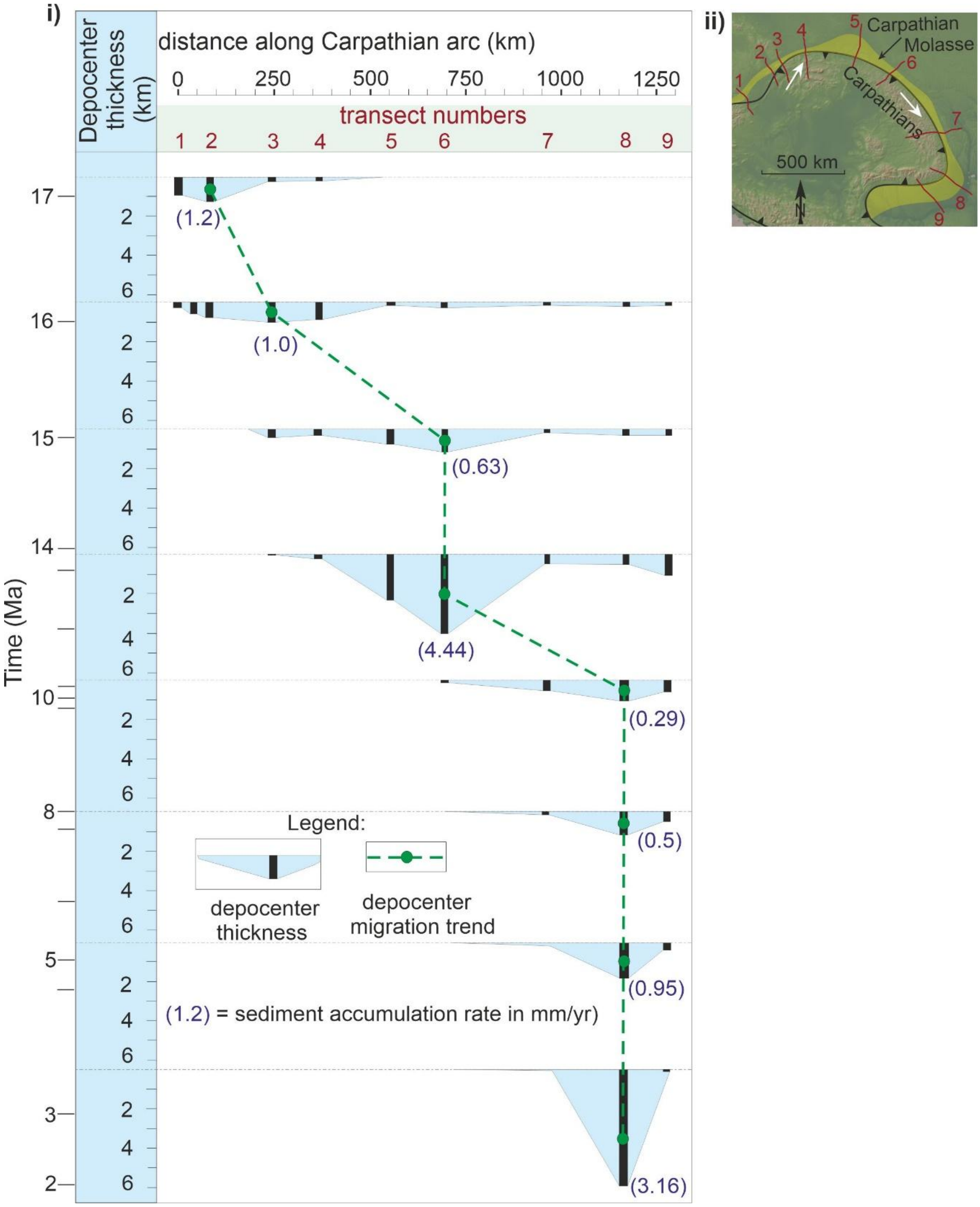

Fig. S15. **Depocenter migration data from Carpathian**. i) thickness of basin depocenter migration interpreted to be influenced by slab tear migration beneath Carpathians (Compiled from ref.[6] datasets). ii) shows the location of transects taken perpendicular to the foreland basin axis where these data were collected (figure made with GeoMapApp[24] (www.geomapapp.org) / CC BY).

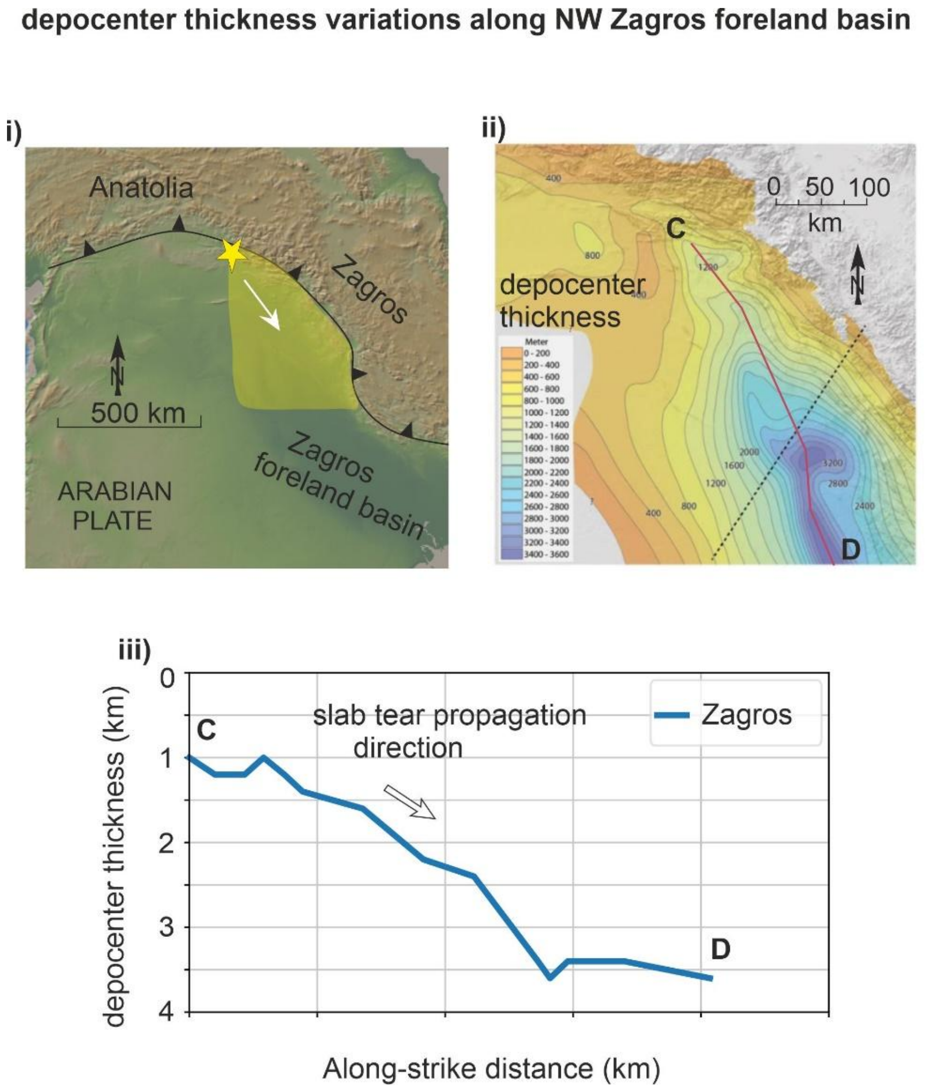

Fig. S16. **Depocenter thickness variations along NW Zagros foreland basin.** (i) Location of the NW Zagros foreland basin, suggested to be affected by slab-tear propagation (figure made with GeoMapApp[24] (www.geomapapp.org) / CC BY). (ii) Location of the foreland basin–parallel transect where basin-depocenter thickness variations, interpreted as the result of slab-tear propagation, were measured (adapted from ref.[11] under CC BY 4.0). (iii) Graphical plot of depocenter thickness variation, showing that thickness increases toward the direction of tear propagation.

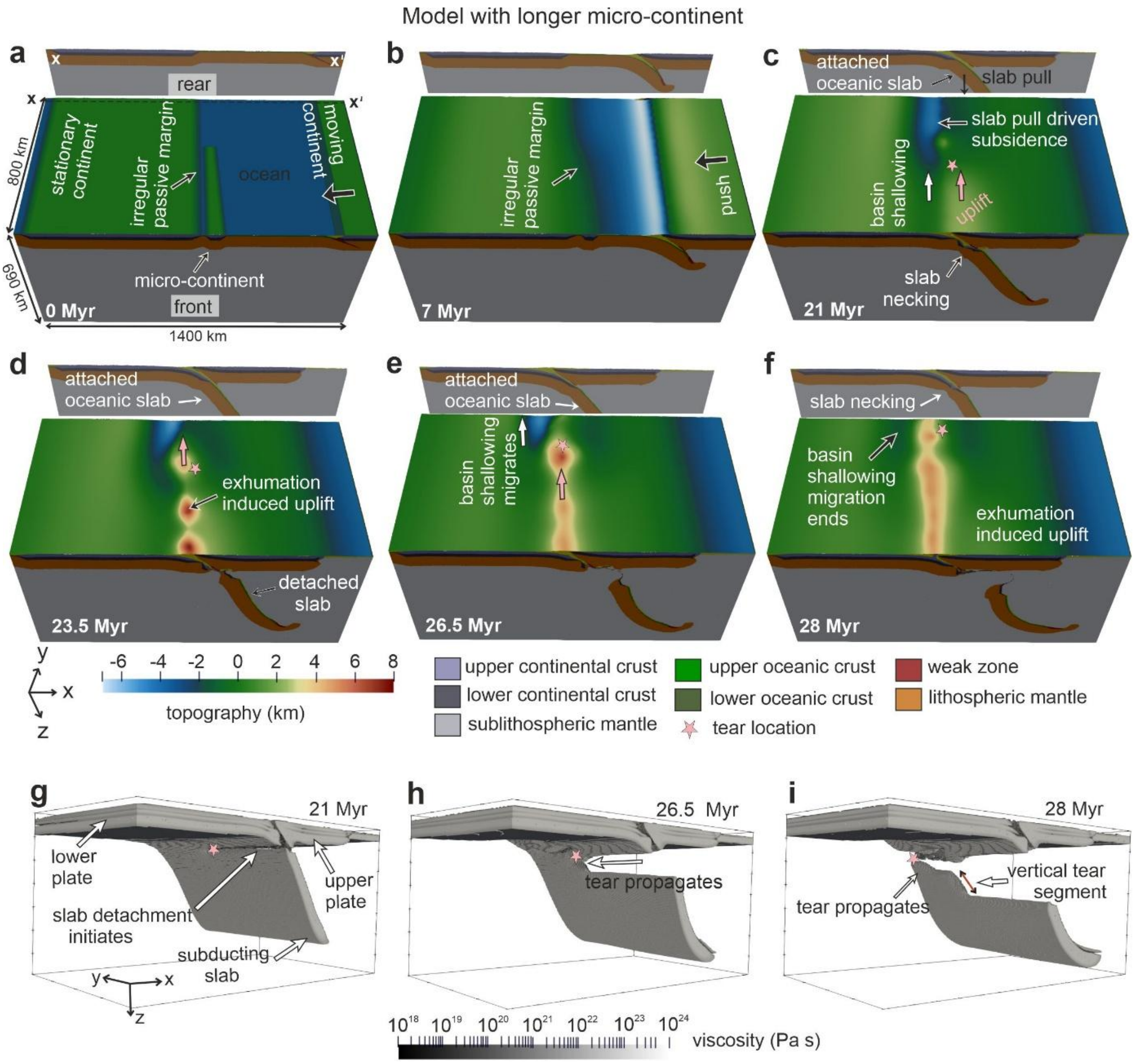

Fig. S17. **Effect of microcontinent extent (y-direction) on slab tearing and surface response**. The reference model contains a 320 km long microcontinent along the y-direction (40% of the model length). Additional model is run with longer microcontinent lengths of 480 km (60% of the model length). The longer-microcontinent model shows slab tearing and propagation similar to the reference model (Model_1).

Model with shorter micro-continent

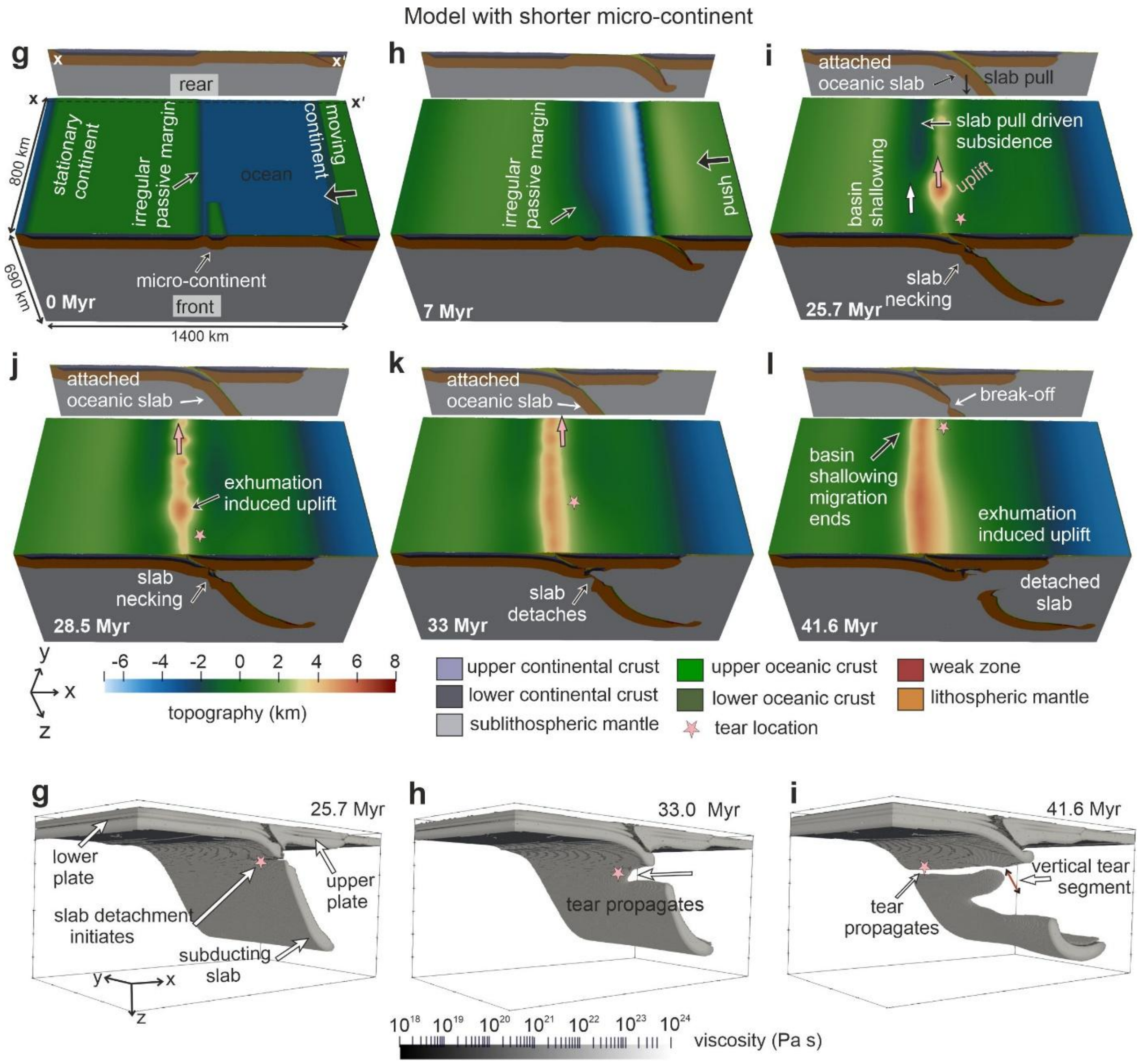

Fig. S18. **Effect of microcontinent extent (y-direction) on slab tearing and surface response.** The reference model (Model_1) contains a 320 km long microcontinent along the y-direction (40% of the model length). Additional model is run with shorter microcontinent length of 160 km (20% of the model length). The model shows slab breakoff initiation takes longer duration, and it delays tear propagation along strike compared to the reference model. But, continued convergence causes collision and topographic growth to begin before tearing reaches the rear end of the model, and slab breakoff there occurs at greater depth than at the front end. As a result, the initial along-strike variation in foreland basin depth, modulated by slab-tearing, is overprinted by collision-related topography growth. This would make the surface expression of slab tearing harder to isolate from collisional overprints.

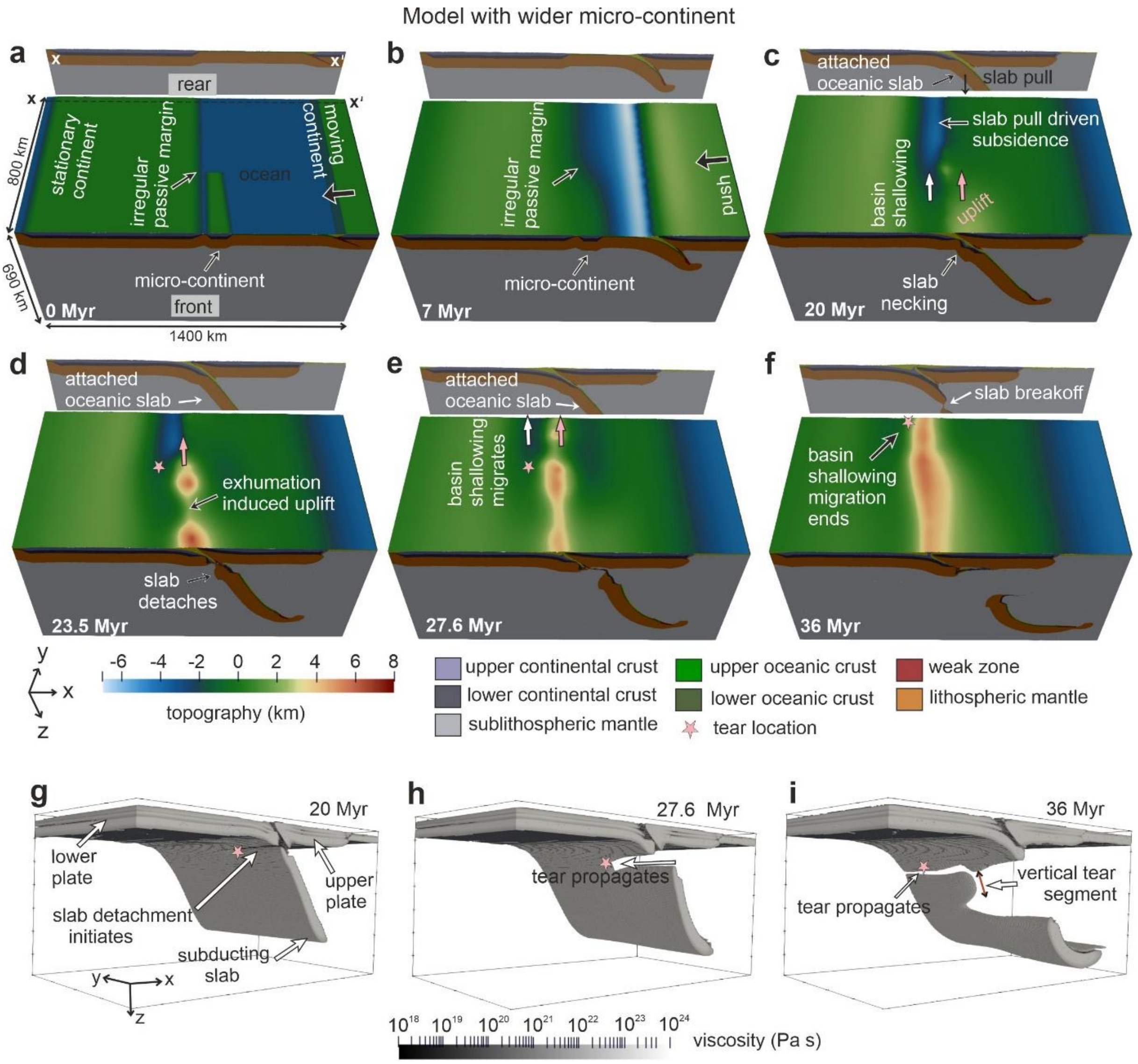

Fig. S19. **Effect of microcontinent width (x-direction) on slab tearing and foreland basin evolution**. In the reference model (Model_1), the microcontinent is 40 km wide in the x-direction. Additional model is run with width of 60 km. Here, slab breakoff at the frontal end begins at the same time as in the reference model (~20 Myr). However, the wider microcontinents delay overall along-strike tear propagation. This is due to a longer-lived phase of vertical tearing at the rear edge of the microcontinent, where the tear transfers from the microcontinental to the oceanic domain. The delayed tear propagation is accompanied by a longer duration of foreland basin subsidence at the rear end of the model.

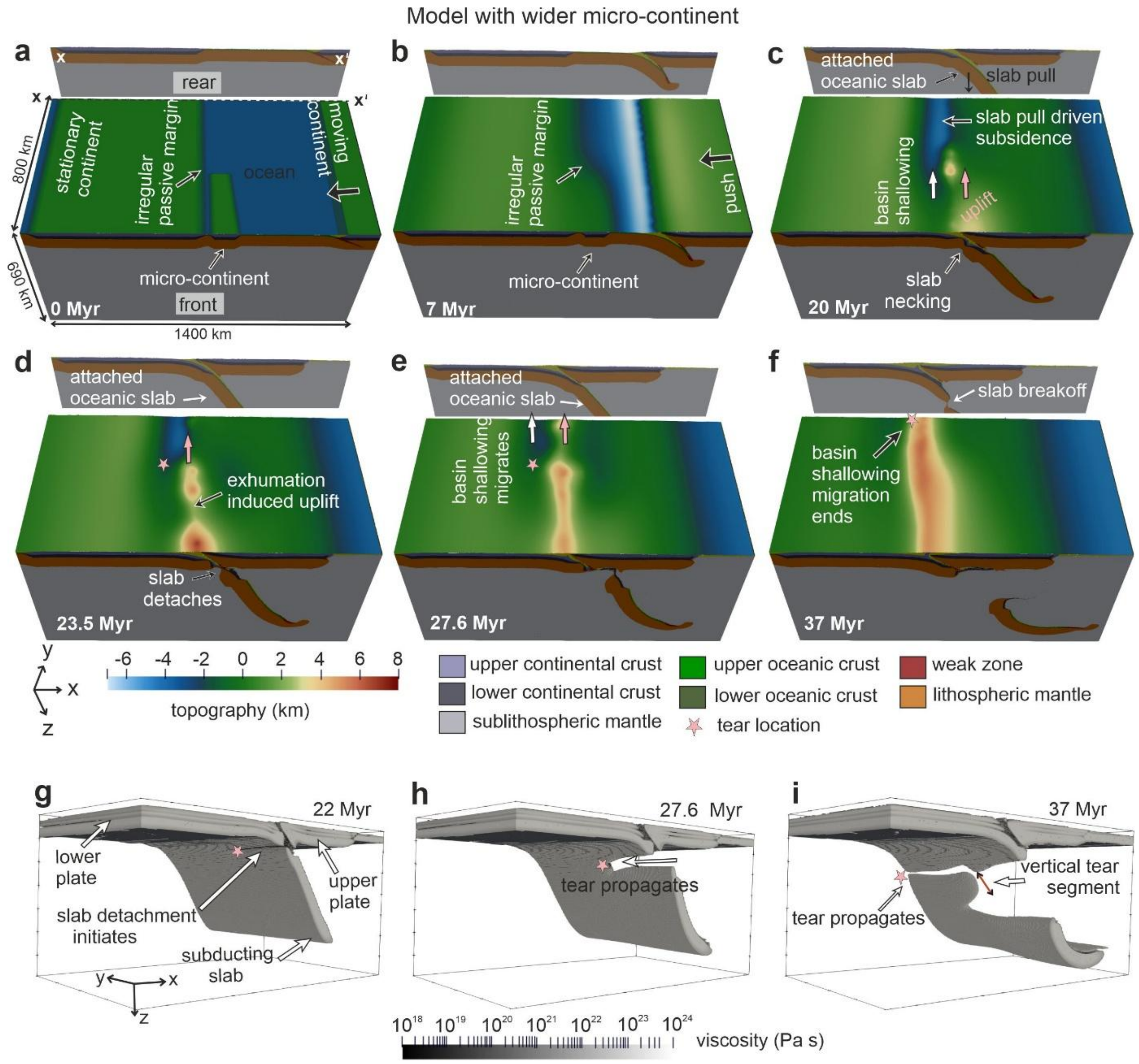

Fig. S20. **Effect of microcontinent width (x-direction) on slab tearing and foreland basin evolution**. In the reference model, the microcontinent is 40 km wide in the x-direction. Additional models is run with width of 80 km. Here, slab breakoff at the frontal end begins at the same time as in the reference model (~20 Myr). However, the wider microcontinents delay along-strike tear propagation. This is due to a longer-lived phase of vertical tearing at the rear edge of the microcontinent, where the tear transfers from the microcontinental to the oceanic domain. The delayed tear propagation is accompanied by a longer duration of foreland basin subsidence at the rear end of the model.

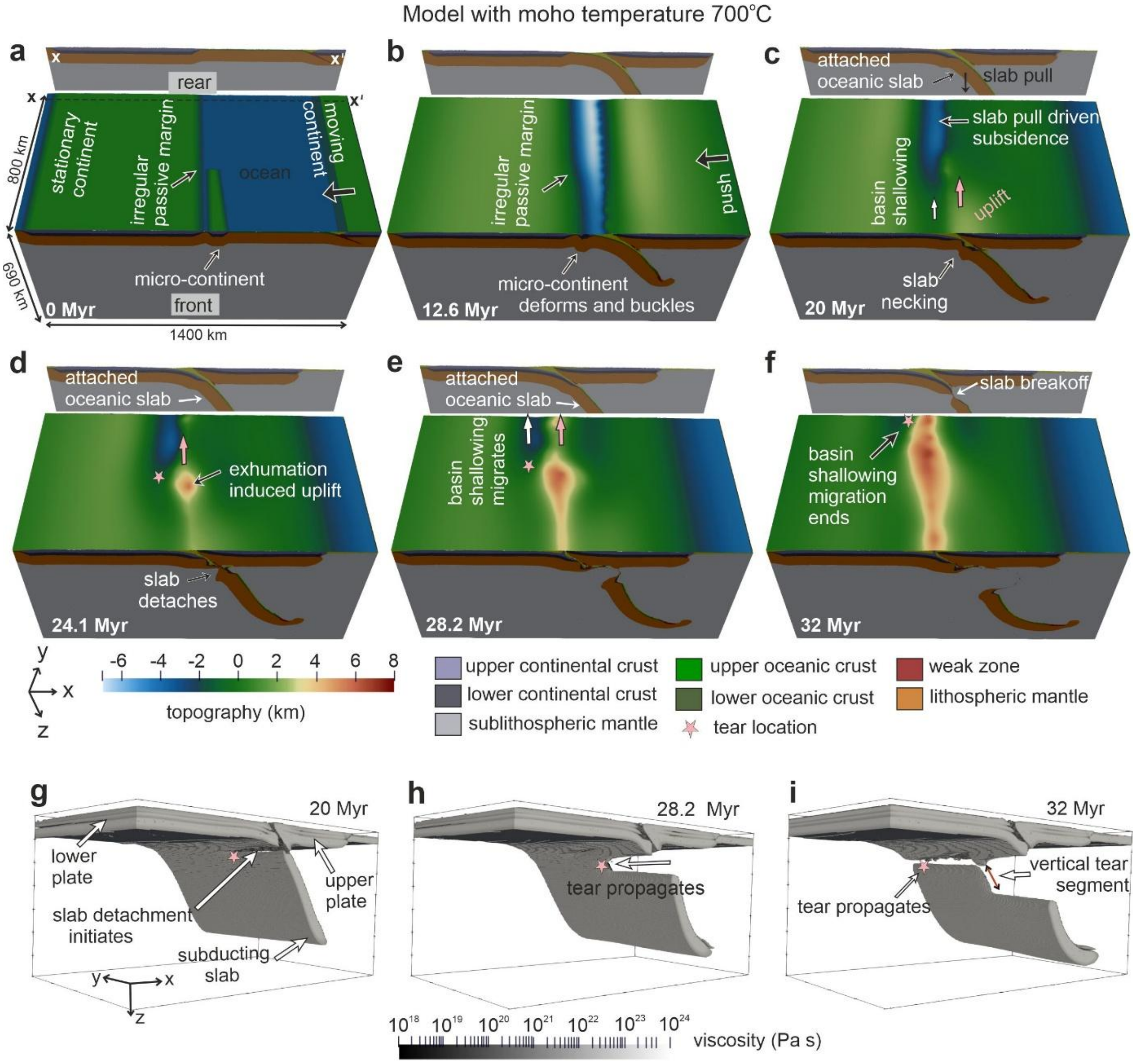

Fig. S21. **Effect of micro-continent thermal structure on slab detachment and tear propagation**. To test how the thermal structure of the micro-continent influences slab detachment and tearing, we varied the Moho temperature within the micro-continent domain. In the reference model, the Moho temperature is 520 °C. We compare this with an additional model in which the Moho temperature is increased to 700 °C. The higher Moho temperature promotes stronger intraplate deformation within the micro-continent and enhances its vertical thickening as it approaches the subduction zone, owing to collision-induced stress transmission. Although slab necking initiates at approximately the same time as in the reference model, breakoff at the frontal end takes longer to complete in the warmer models. As a result, along-strike tear propagation towards the rear end is delayed. In the reference model, the tear reaches the rear end at ~28 Myr, whereas in the models with elevated Moho temperature it reaches the rear end at ~32 Myr.

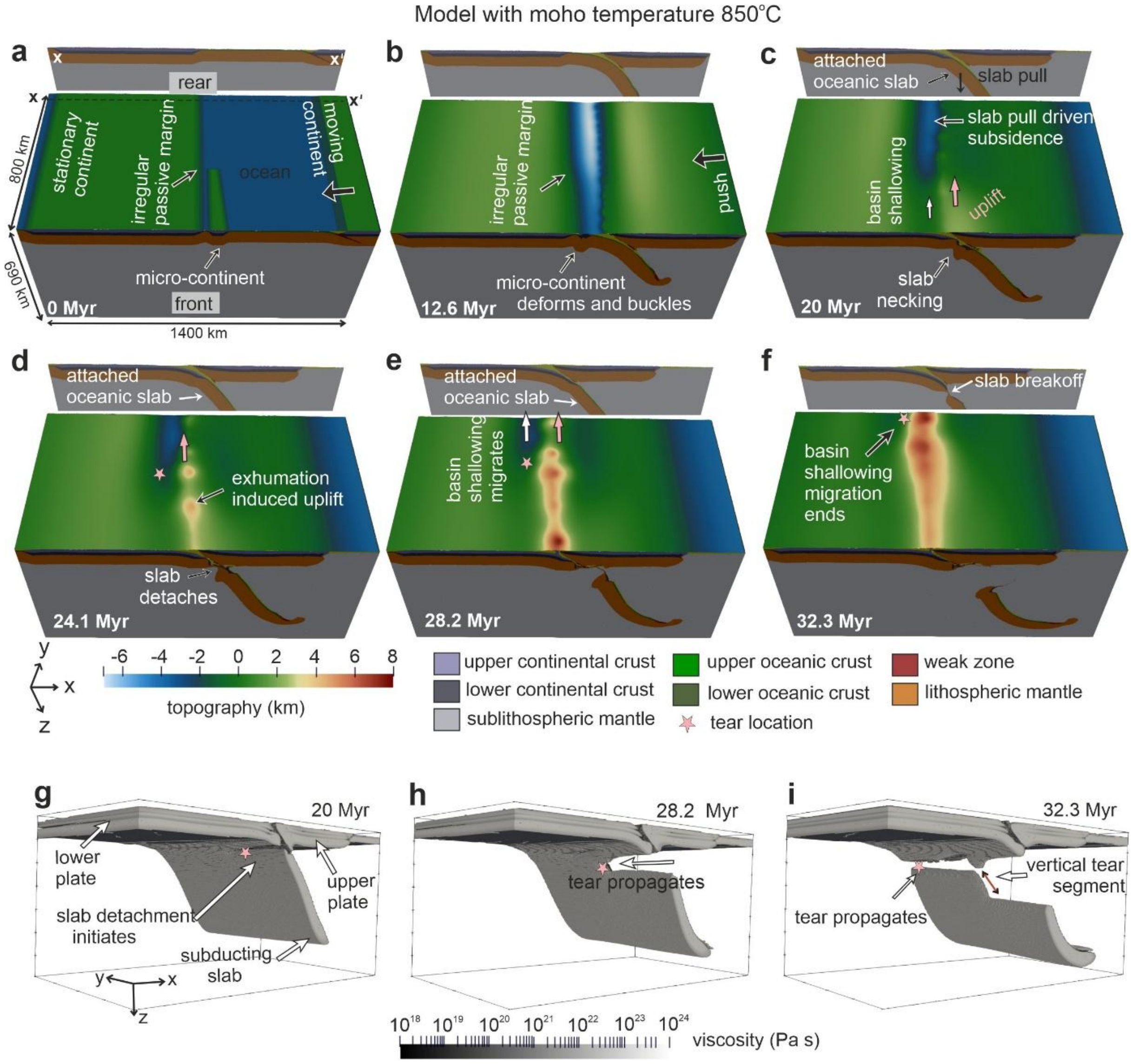

Fig. S22. **Effect of micro-continent thermal structure on slab detachment and tear propagation.** We further increased the Moho temperature from the reference 520°C to 850 °C. The model shows similar evolution like in model with Moho temperature of 700°C (see Figure S21).

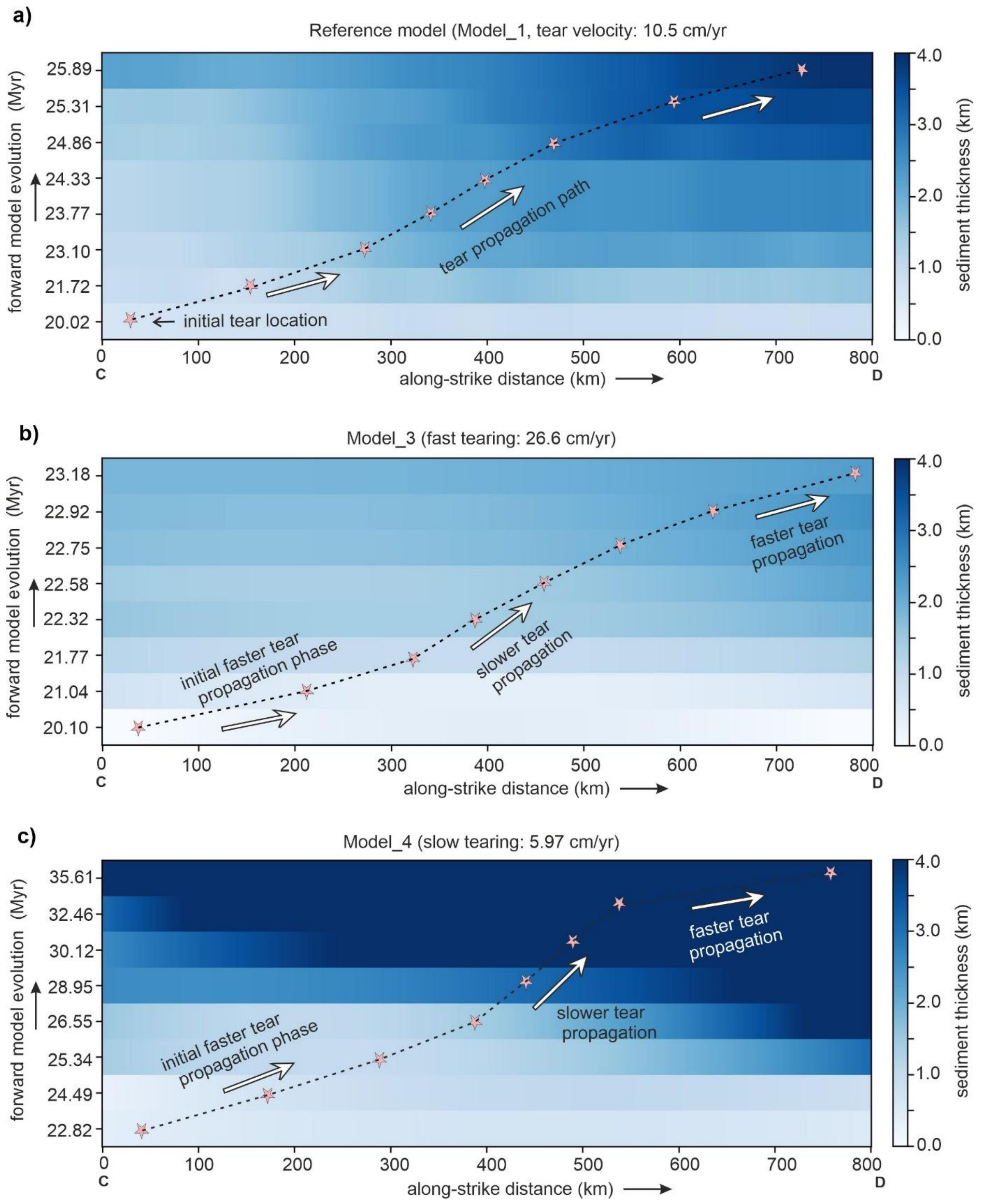

Fig. S23. **Sediment-thickness variations along the foreland basin for different slab-tear propagation rates**. (a) Reference model. (b) Model with the fastest slab-tear propagation. (c) Model with the slowest slab-tear propagation. In all panels, sediment thickness is plotted as a function of along-strike distance of the foreland basin (x-axis) and time (y-axis). The figure illustrates that slow tear propagation results in more pronounced and longer-lasting along-strike sediment-thickness variations, whereas fast tear propagation produces weaker and shorter-lived variations. Models with intermediate tear-propagation rates will show the same general relationship, where the magnitude and duration of along-strike sediment-thickness differences will depend on tear-propagation duration and velocity. Note that the overall along-strike tear-propagation velocity decreases during the vertical tearing phase, but accelerates during the terminal phase due to increased slab-pull forces from the hanging slab (Fig. S3).

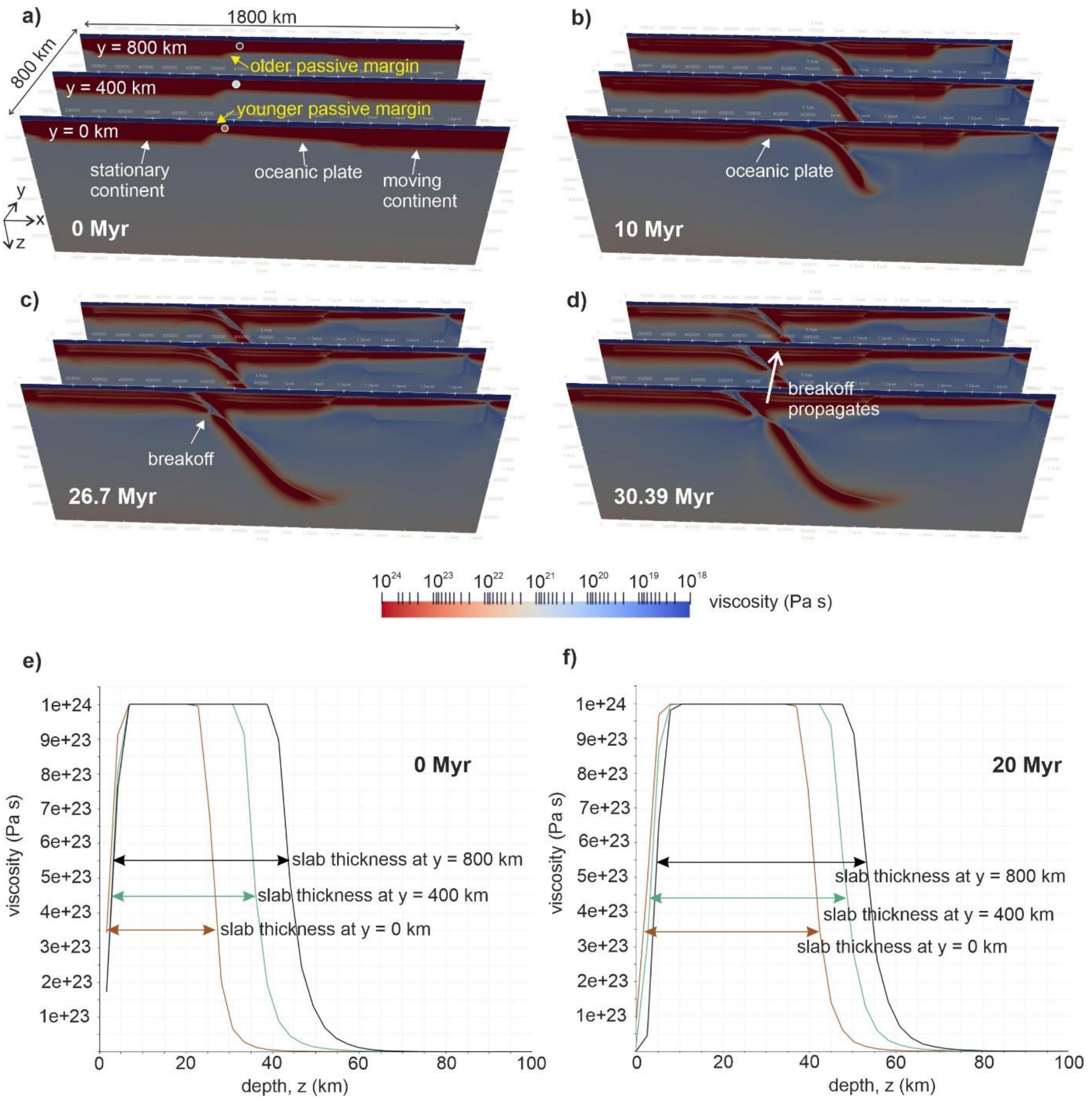

Fig. S24. **Evolution of effective viscosity of subducting oceanic lithosphere in the Model-5**. At the frontal section (y = 0 km) the initial age of the oceanic slab is assigned 10 Myr, whereas at the rear side (y = 800 km) it is assigned 40 Myr. The thermal age difference between oceanic domains results in an along-strike variation in slab thickness (shown by effective viscosity plot in panels **e** and **f**). We measured slab thickness at the start of the model run (Panel **e**) and before onset of slab tearing (Panel **f**) at three points located along the passive margin (shown by circled dots in Panel **a**). Although the slab thickens during model evolution, the relative along-strike thickness contrast still remains up to the onset of tearing. Note that 5e+23 Pa s means 5×10^{23} Pa s.

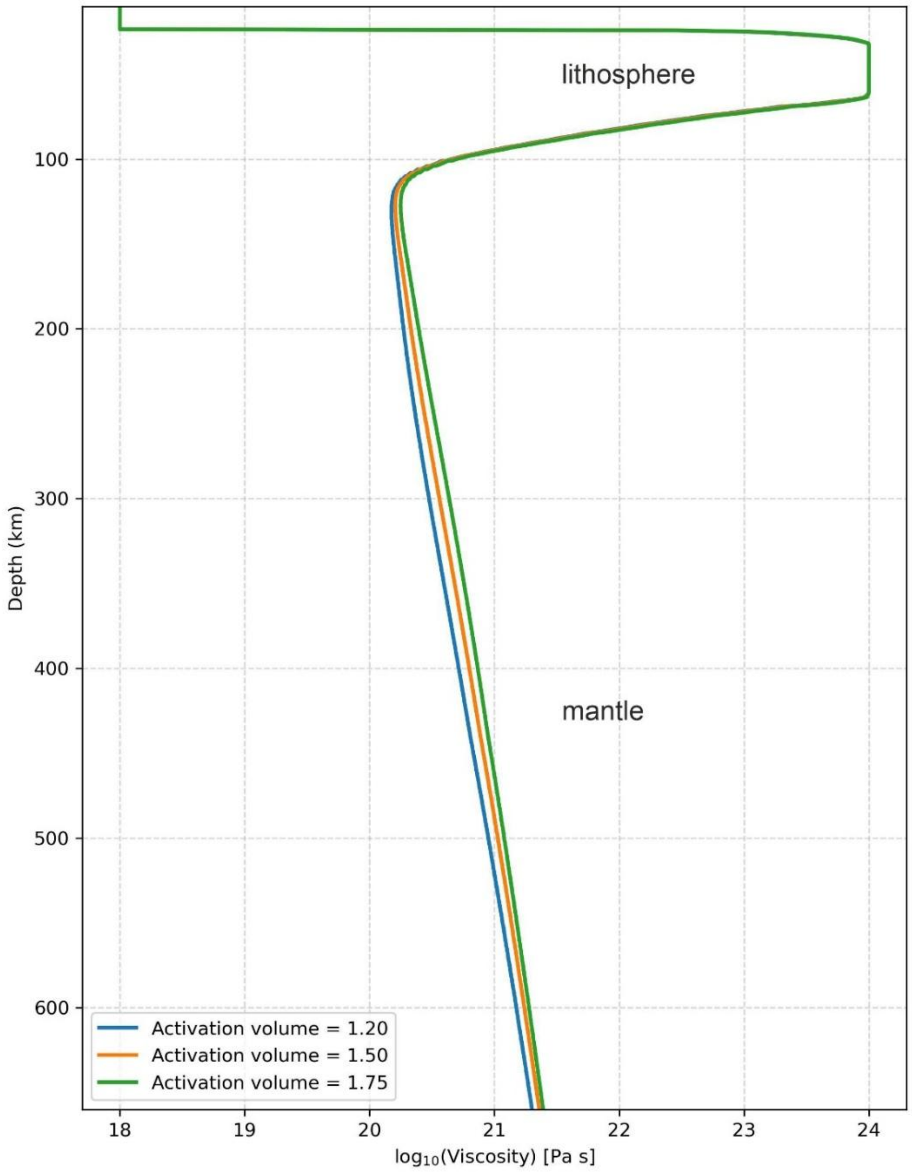

Fig. S25. **Comparison of viscosity with depth for different activation volume of the mantle rocks.** See Supplementary Table S3, it is measured at 5 Myr of the model run. Increasing activation volume leads to higher mantle viscosity. As a result, although slab tear occurs at relatively shallow upper-mantle depths, the deeper part of the oceanic slab is more strongly supported by the surrounding high-viscosity mantle. This slows slab sinking and therefore reduces tear propagation velocity.
